# Supplementary material for: Risk Factors Associated With Mastitis in Smallholder Dairy Farms in Southeast Brazil
Source: Animals (Basel). 2021 Jul 14;11(7):2089. doi: 10.3390/ani11072089 (PMC8300378; doi:10.3390/ani11072089)
Supplement: Supplementary file 1 [file animals-11-02089-s001.zip › animals-1238639-supplementary.pdf]

Risk factors associated with mastitis in smallholder dairy farms in southeast Brazil.

Aline Callegari Silva[a] *, Richard. A. Laven[b], Nilson Roberti Benites[a]

a Department of Preventive Veterinary Medicine and Animal Health, Faculty of Veterinary Medicine and Animal Science, University of São Paulo (USP), Av. Prof. Dr. Orlando Marques de Paiva, 87, CEP 05508-270 São Paulo, Brazil.
b School of Veterinary Science, Massey University, Palmerston North, 4442, New Zealand

*Corresponding author: Aline Callegari Silva email: callegari@alumni.usp.br
ORCID: https://orcid.org/0000-0002-0445-5622

Appendix – Questionnaire of characterization of productive systems.

General Data

Date:
Address::
Farmer name:
Contact::
E-mail:

Property Information

1.What is the size of the property (ha)?
2. What is the area of permanent protection area (APP) and Legal Reserve (LR) in ha?
3. What is the total area, how much is destined for dairy activity (ha)?
5. What is the available grazing area (ha)?
6. Type of system
_ grazing _semiconfined _ confined _ other
7.Production Model
_Conventional
_ Transition to organic
_ Organic

Characterization of family or contracted labor.

1. Indicate who conducts the animals, time spent, monthly salary (R$/Month):
2. Indicate who milks the cows, time spent, monthly salary (R$/Month):
3. Indicate who does the administration, time spent, monthly salary (R$/Month):
4. Indicate who does the administration, time spent, monthly salary (R$/Month):
5. Indicate who does the health management time spent, monthly salary (R$/Month):

Herd Characteristics

1. Number of animals in dairy farming:
2. Number of lactating cows:
3. Number of primiparous lactating cows:
4. Number of multiparous lactating cows:
5. Number of dry cows:
6. Number of heifers (12-24 months):
7. Number of heifers (> 24 months):
9. Number of calves weaned:
10. Number of calves suckling:
11. Age of weaning calves
13. Number of bulls:
14. Stoking rate of total milk area:
15. Stoking rate of pasture area:

Reproduction and milk production

1. Strategy used for breeding the herd:
- Artificial insemination
-Use of bull
-Transfer of embryos
2. What interval between deliveries?
3. What is the main breed on the farm?
-Holstein
-Jersey
-Crossbreed Gir X Holstein (Girolando)
-Crossbreed

Milk Production

1. Total milk production (liters/month):
2. Average productivity of cows (liters/cow):
3. Average milk price(R$/liter):
4. Bonus on the value of milk (R$/liter):
5. Quantity of milk discarded in the last month/day or month:
6. Quantity of milk supplied to calves in the last month/day or month:
7. Milk quality in the last month (tank milk):
Somatic cell count (CCS):
Total bacterial count in milk (CBT):

Herd Health

1. Major diseases affect the herd in recent months?
2. Performs vaccination for:

-Foot and Mouth disease
-Brucellosis
-Carbuncle
-IBR / BVD
- Bovine Pasteurellosis/ Paratyphoid
Enterotoxemia/colibacillosis
-Rabies
-Mastitis

Mastitis in dairy cows

1. Perform treatment of cases with:
   - Clinical Mastitis
   - Subclinical Mastitis
   - Variable

2. How many clinical cases did not treat in the last 3 months and why?
3.In which situation does not treat clinical mastitis?
4. Which medications utilize to treat mastitis:
-Antibiotic tube:
- Systemic Antibiotic:
-Anti-inflammatory:
- Dry cow antibiotic:
-Homeopathy:
-Other:
5. Describe mean of treatment time per case and disposal milk days per case:
6. Mean of time (min) and expenses with veterinary per clinical cases (R$/mastitis cases)?
7. How many cases have you had veterinary assistance in the last 3 months?
8. How many cases did the animals dry up due to mastitis in the last 3 months?
9. Did you have animal disposal?
10. Did you treat subclinical mastitis?
11. Value in milk price penalty for high CCS level:

Preventive Measures

1. What are the preventive measures used in the property and in the time spent (min/day)?
-Milking clinical cases in the end
-Milking subclinical cases in the end
-Pré-dipping
-Pós-dipping
-Wash dirty teat
-Wash dirty udder
-Wash milking liners between milking
-Wash milking liners end milking
-Use separate clothe or paper
- Clean milking parlor
- Milking Maintenance
- Gloves
-Treat dry cows
-Vaccine
- Protocol treatment by veterinary
-Keep cows standing after milking
-Provide mineral supplement specific to dry cows
-Homeopathic salt
-Optimize feed before calving (how many days before calving)
-Reduce overcrowding
-Clean stables
2. Time and expenses with preventive measures (R$/Month):
3. What mastitis control measures are being used on the property and frequency (weekly, biweekly, or monthly):
- Antibiogram and culture.
- Somatic cells account (SCC), bacterial total account (BTC), and tank milk composition analysis
- Individual SCC
- California mastitis test (CMT)
4. Average of expenses with control measures(R$/ Monthly):

Food management

1.There is a specific nutritional balance for your property?
2. What foods are provided to the animals and quantity (kg/animal/day) in summer and winter.
-Pasture
- Ration
-Silage
-Ray
-Barley
-Sugar Cane
-Other types:
3. Time of pasture access:
-Summer:
-Winter:
4. Perennial grazing area(ha) (fixed):
- Total cost of pasture formation and/or annual maintenance (R$/annual):
5. Annual summer pasture area (ha):
- Total cost of pasture formation and/or annual maintenance (R$/annual):
6. Annual winter pasture area (ha):
- Total cost of pasture formation and/or annual maintenance:

7. Silage production area (ha):
-Total production cost (R$/annual):
8. Hay production area (ha):
-Total Production Cost (R$/annual):
9. Production area of another crop (ha)
-Observations regarding the area and cost of production and / maintenance:
10. Machine time used for feeding(hour/day):
11. Mineral supplement offer. Quantity (grams/day) in lactating cows.

Dry cow Management

1. The decision to dry cow is based in:
-Gestation time
-Decrease n milk yield
-Score condition of the animal
-Lactation time
2. How many days before calving dry the cows?
- 60 days
- 45 days
- <45 days
-Don't use any criteria
3. What strategy do you use to dry cows?
-Abrupt
-Gradual
-Use of intra-mammary antibiotic
-Use of parenteral antibiotic
-Use of sealant
-Stop doing milking only
-Remove the feed
-Does nothing (wait for the cow to dry on its own)
4.-Reduces the supply of food to dry cows?
-Yes
-No
-Sim
5.What type of salt is offered to dry cows?
-Specific salt for dry cow
-The same as lactating cows
-Other

Prepartum and Postpartum Management

1. Is there a specific location for prepartum ?
-Yes
-No
2. When the gestation cows were conduced to this prepartum place?
- One week prepartum
- < one week prepartum
- > one week prepartum
- NA
3. Which food is provided to prepartum cows?
-Pasture
-Silage
-Ration
4. After calving the cow's food change?
-Yes
-No
5. What changed in the food of post postpartum cows?
- Increase the ration (Kg/cow/month)
-Increase silage (Kg/cow/month)
6.How long does the calves stay with the cow?

Milking Management

1. What is the average distance that cows go through from stalls to milking parlor (m)?
2 Time spend to arrive at milking parlor:
4. Time for start milking
5. Time for finalize milking:
6. Describe the waiting pen, clean, type of floors, presence of cover and trough;
7. Describe the exit pen, cleanness type of floors, presence of cover and trough;

Milking Parlor Environment

1. Describe the milking parlor as to cleanness, type of floor, and cover:
2.Type of milking parlor:
-Fishbone
-Tandem type (the single row with side exit)
-Parallel (animal position: perpendicular to the pit)
-Line
-Parallel without pit (conventional stable)
3. How many clusters of liners are used in the milking:
4. Number of milking/day:
5. Mean number of cows is milking simultaneously:
